# Different Frontal Involvement in ALS and PLS Revealed by Stroop Event-Related Potentials and Reaction Times

**DOI:** 10.3389/fnagi.2013.00082

**Published:** 2013-12-12

**Authors:** Ninfa Amato, Nilo Riva, Marco Cursi, Ana Martins-Silva, Vittorio Martinelli, Mauro Comola, Raffaella Fazio, Giancarlo Comi, Letizia Leocani

**Affiliations:** ^1^Neurological Department, Institute of Experimental Neurology (INSPE), Scientific Institute Hospital San Raffaele, University Vita-Salute San Raffaele, Milan, Italy

**Keywords:** ALS, PLS, cognitive impairment, ERP, Stroop task, executive function

## Abstract

**Background**: A growing body of evidence suggests a link between cognitive and pathological changes in amyotrophic lateral sclerosis (ALS) and in frontotemporal lobar degeneration (FTLD). Cognitive deficits have been investigated much less extensively in primary lateral sclerosis (PLS) than in ALS.

**Objective**: To investigate bioelectrical activity to Stroop test, assessing frontal function, in ALS, PLS, and control groups.

**Methods**: Thirty-two non-demented ALS patients, 10 non-demented PLS patients, and 27 healthy subjects were included. Twenty-nine electroencephalography channels with binaural reference were recorded during covert Stroop task performance, involving mental discrimination of the stimuli and not vocal or motor response. Group effects on event-related potentials (ERPs) latency were analyzed using statistical multivariate analysis. Topographic analysis was performed using low-resolution brain electromagnetic tomography (LORETA).

**Results**: Amyotrophic lateral sclerosis patients committed more errors in the execution of the task but they were not slower, whereas PLS patients did not show reduced accuracy, despite a slowing of reaction times (RTs). The main ERP components were delayed in ALS, but not in PLS, compared with controls. Moreover, RTs speed but not ERP latency correlated with clinical scores. ALS had decreased frontotemporal activity in the P2, P3, and N4 time windows compared to controls.

**Conclusion**: These findings suggest a different pattern of psychophysiological involvement in ALS compared with PLS. The former is increasingly recognized to be a multisystems disorder, with a spectrum of executive and behavioral impairments reflecting frontotemporal dysfunction. The latter seems to mainly involve the motor system, with largely spared cognitive functions. Moreover, our results suggest that the covert version of the Stroop task used in the present study, may be useful to assess cognitive state in the very advanced stage of the disease, when other cognitive tasks are not applicable.

## Introduction

Amyotrophic lateral sclerosis (ALS) is the most common form of motor neuron disease (MND). The dysfunction of higher cognitive abilities, from mild cognitive impairment to frank dementia, is common in ALS (Ringholz et al., 2005), with a prevalence that ranges from 10 to 75% in patients (Miller et al., 2009), mainly involving cognitive and behavioral frontotemporal functions (Strong et al., 2009). In recent years, a growing body of evidence (Neary et al., 2000; Lomen-Hoerth et al., 2003; Rippon et al., 2006; Murphy et al., 2007) has suggested a link between ALS and frontotemporal lobar degeneration (FTLD).

Previous studies that assessed subjects at rest (Kew et al., 1993; Abe et al., 1997) or during the performance of executive tasks (Ludolph et al., 1992; Abrahams et al., 1995) reported a significant decrease in frontal lobe activation in non-demented ALS patients who presented cognitive deficits.

Gray matter and white matter changes that involve frontal and temporal lobes, particularly the anterior cingulate cortex (ACC), revealed by magnetic resonance imaging (MRI), have been reported in ALS-FTLD patients (Lillo et al., 2012). Moreover, decreased connectivity of the frontal cortex and compensatory increased connectivity of the parietal cortex, which plays a role in the effort to maintain cognitive efficiency, have been reported in ALS patients (Agosta et al., 2013).

Remaining unclear, however, is whether considerations of frontotemporal syndromes in ALS can be applied to other MND variants, such as primary lateral sclerosis (PLS). Cognitive deficits in PLS have been investigated much less extensively than cognitive deficits in ALS. Previous studies demonstrated that PLS patients presented frontotemporal impairments that were qualitatively similar to cognitive dysfunction in ALS patients (Caselli et al., 1995; Piquard et al., 2006; Murphy et al., 2008) and in a proportion of patients that was comparable to ALS (Grace et al., 2011). However, these have not been consistent features. Other authors have reported normal intellectual function in PLS (Russo, 1982; Pringle et al., 1992).

Abnormalities in the regional distribution of cerebral blood flow (rCBF) localized in the precentral gyrus and ACC have been reported (Le Forestier et al., 2001). Similar abnormalities have been documented in ALS (Abrahams et al., 1996).

Other authors reported different cerebral involvement in PLS patients compared with ALS patients. PLS patients presented lower fractional anisotropy (FA) in the body of the corpus callosum and white matter adjacent to the right primary motor cortex. ALS patients, in contrast, presented a reduction of FA in white matter adjacent to the superior frontal gyrus (Ciccarelli et al., 2009).

While electrophysiological abnormalities to motor tasks have been documented both in PLS (Bai et al., 2006) and ALS (Westphal et al., 1998; Inuggi et al., 2011; Riva et al., 2012), few neurophysiological studies have investigated electrophysiological correlates of executive function in the two disease forms. Studies assessing event-related potentials-ERPs in an oddball paradigm in ALS found a delay or decreased amplitude of P3, which reflects the orienting response, i.e., an involuntary shift of attention to new, unexpected, or unpredictable stimuli (Vieregge et al., 1999; Hanagasi et al., 2002; Paulus et al., 2002; Ogawa et al., 2009), which was associated with low scores on neuropsychological tests that assess attention and executive function (Paulus et al., 2002) and disease duration and severity (Raggi et al., 2008; Volpato et al., 2010).

Further ERP studies that utilize cognitive tasks with PLS patients are needed to better clarify whether PLS spares cognitive function and manifests predominantly with motor symptoms or whether cognitive involvement in PLS patients is similar to ALS patients who present especially with executive dysfunction (Grace et al., 2011).

Among the cognitive tasks suitable for ERP analysis, the Stroop task (Stroop, 1935), which assesses action-monitoring function (i.e., the ability to process competing information and response inhibition), could be a good candidate and has already been widely applied in the study of executive functions that are particularly compromised in ALS patients.

Previous studies investigated ERPs with the Stroop task in healthy subjects and neurological and psychiatric disorders (Liotti et al., 2000; Annovazzi et al., 2004; Markela-Lerenc et al., 2004; Badzakova-Trajcov et al., 2009; Kikuchi et al., 2012), supporting the role of prefrontal regions, specifically the ACC, in the executive control necessary for conflict resolution during task performance.

A central role for the ACC in the execution of the Stroop task has been reported by most neuroimaging studies (Milham et al., 2001; van Veen et al., 2004; Milham and Banich, 2005; van Veen and Carter, 2005) and combined ERP-functional MRI (fMRI) studies (Gonzalez-Rosa et al., 2013).

Goldstein et al. (2011) used fMRI to investigate ALS patient performance in the Stroop task and negative priming task (i.e., a further adaptation of the Stroop task). The authors reported an increase in the activation of the left middle and superior temporal gyrus and ACC during Stroop task performance. The results suggested greater difficulty suppressing word reading in ALS patients. They also documented a decrease in the activity of the left cingulate gyrus, left precentral gyrus, and left medial frontal gyrus during negative priming task performance. According to the authors, this accounted for less difficult reactivation of the previously suppressed stimulus because of less efficient response inhibition.

The present study evaluated bioelectrical activity during a modified Stroop task that did not require a motor or vocal response in non-demented ALS patients, non-demented PLS patients, and healthy control subjects. To disentangle stimulus processing from motor activity, thus better clarifying the degree of cognitive involvement in PLS with respect to ALS, mental discrimination was used to avoid the impact of speech or movement impairments on the results.

## Materials and Methods

### Subjects

Patients were consecutively recruited from our Neurological Department, including 32 non-demented ALS patients (21 males, 11 females; mean age, 57 years) and 10 non-demented PLS patients (one male, nine females; mean age, 52 years; Table 1). The clinical diagnosis was based on the El Escorial revised criteria and included patients with laboratory-supported probable, probable, and definite ALS (Brooks et al., 2000). PLS was diagnosed according to Pringle’s criteria (Pringle et al., 1992). Patients with a current or past history of neurologic disease other than MND, with other neuropsychiatric or medical disorders, or who used psychoactive drugs within the prior 2 months were excluded. Clinically healthy control subjects had a similar age and sex distribution (12 males, 15 females; mean age, 57 years), a normal neurologic examination, and no history of neurologic or psychiatric disorders. The participants provided informed consent to participate in the study, which was approved by the local ethics committee.

**Table 1:** **Demographic and clinical characteristics of ALS and PLS patients [mean values (Standard Deviation)]**.

Diagnosis	Age	Gender	Disease duration (months)*	UMN score	ALS∖FRS	Bulbar score	MRC total score	Disease progression rate
ALS	57 (9)	M:21-F:11	20.8 (19)	9 (5.8)	32.5 (3.6)	11.03 (1.4)	101.1 (13.6)	0.66 (0.75)
PLS	52 (11)	M:1-F:9	45.9 (38)	14 (2.7)	33.5 (4.5)	10.5 (1.4)	117.5 (7.9)	0.18 (0.14)

M, males; F, females; UMN, upper motor neuron; ALS∖FRS, amyotrophic lateral sclerosis/functional rating scale; MRC, Medical Research Council scale. *Disease duration at the time of testing (a diagnosis of PLS was confirmed in participants with a disease duration <36 months at the time of testing).

### Clinical assessment

Demographic data and detailed neurological findings were recorded. Disease severity was estimated using the ALS Functional Rating Scale (ALS-FRS; The ALS CNTF Treatment Study Phase I-II Study Group, 1996). The patients were graded in terms of upper motor neuron (UMN) “burden” by totaling the number of pathological UMN signs upon examination. These were taken as pathologically brisk biceps, supinator, triceps, finger, knee, and ankle reflexes, and extensor plantar responses assessed bilaterally and brisk facial and jaw jerks (range: 0–16; Turner et al., 2004). Disease duration at the time of the investigation was calculated in months, from the date of first symptoms to the date of examination. The rate of disease progression was calculated using the following formula (Ciccarelli et al., 2006): *disease progression rate* = (*40* – *ALS-FRS score*)/*disease duration*.

Muscle strength was graded on the Medical Research Council (MRC) scale, from 0 to 5, in selected upper and lower limb (LL) muscle groups. On each side, seven upper limb (UL) muscles and five LL muscles were tested as previously described (de Carvalho et al., 2003). Three scores were obtained: UL-MRC sum score (maximum: 70), LL-MRC sum score (maximum: 50), and total MRC sum score (UL + LL MN score; maximum: 120).

Patients with other neuropsychiatric or medical disorders or who used psychoactive drugs during the previous 2 months were excluded.

### Stroop reaction times

Reaction times (RTs) in the Stroop task were measured using a computerized version implemented in commercial STIM software (Neuroscan, Herndon, VA, USA). Responses were recorded using a computer mouse with two response buttons. Four color words (green, red, yellow, and blue) written in congruent (50%) or incongruent (50%) color were randomly presented (stimulus duration, 200 ms; intertrial interval, 3.5 s) in four different series of 32 stimuli each.

In the first condition (simple RT – SRT), the subjects had to press a button for every stimulus presentation, regardless of stimulus type. The second condition (go/no-go RT) consisted of two series, in which a response was required to either the incongruent (go/no-go I) or congruent (go/no-go C) stimuli. In the third condition (choice RT), the subjects had to press one button after the congruent stimuli (choice C) and the other button after the incongruent stimuli (choice I). For each series, the response latency was measured only for correct responses. Trials with latencies that exceeded 2.3 s were considered omissions and excluded from the calculation of average RTs and accuracy. The latter was calculated in the complex RTs (go/no-go and choice) as the percentage of correct responses.

### Event-related potential recording

Twenty-nine electroencephalography (EEG) channels with binaural reference were recorded using scalp electrodes set on an elastic cap (Electrocap International, Eaton, OH, USA). The EEG signal was amplified (Synamps, Neuroscan, Herndon, VA, USA), filtered (DC–50 Hz), and digitized (sampling frequency, 250 Hz). The electrooculogram and electromyogram of the right and left extensor pollicis brevis were also recorded to detect eye movements and relaxation failure.

A series of 120 of the same Stroop stimuli used for the RT measurement were presented (stimulus duration, 200 ms; intertrial interval, 6 s). The subjects were instructed to mentally discriminate between congruent and incongruent stimuli. This condition was chosen for ERP recording to avoid movement interference. Attention was monitored by randomly asking the subjects in every 10–15 trials to verbally define the congruency of the last stimulus presented.

### Event-related potential analysis

Epochs from −500 to 1200 ms from stimulus onset were obtained. Linear detrending was performed over the entire epoch to correct for DC drifts. The baseline was then corrected between −500 and 0 ms. Epochs that contained artifacts or muscle relaxation failure upon visual inspection were excluded from the analysis. Initially, separate averages were obtained for congruent and incongruent stimuli. After a preliminary comparison between and within groups, which did not show significant differences between the parameters obtained in the two conditions, data from the congruent and incongruent trials were collapsed into a single ERP for each subject to reduce signal noise.

The latency of the main ERP components [i.e., N1 (O1 or O2 electrode), P2, N2, P3, N4, and LPC (late positive complex, peaking 600–700 ms post-stimulus onset; Fz electrode)] was measured for each subject. The amplitude and topographic analysis was performed at time intervals of the same components (time intervals = group mean latency value of each component ±30 ms) using low-resolution brain electromagnetic tomography (LORETA; Pascual-Marqui et al., 1994; Pascual-Marqui, 1999; see Statistical Analysis below).

### Statistical analysis

The significance of group effects with regard to the number of correct responses (in the choice condition, go/no-go C condition, and go/no-go I condition), RT latency in the choice C condition, choice I condition, go/no-go C condition, go/no-go I condition, and simple RT condition, and latency of the main ERP components (N1, P2, N2, P3, N4, and LPC) was tested using three separate multivariate analyses of variance (MANOVAs). *Post hoc* tests were performed using Bonferroni correction. Correlations between clinical scales and RTs and between clinical scales and ERP latencies were also performed using Spearman’s coefficient for interval scales and Pearson’s coefficient for ordinal scales. All of the statistical tests were performed using SPSS 17 software (Technologies, Chicago, IL, USA). Group differences in the amplitude and topography of ERP waveforms were investigated using LORETA with a statistical non-parametric voxel-wise comparison between the ALS, PLS, and control groups. The level of significance was set at *p* < 0.05.

## Results

### Patients

In the ALS patient group, the mean scores were the following: mean UMN score: 9; mean ALS∖FRS rate: 32.5; mean bulbar score: 11.03; mean MRC total score: 101.1; mean disease progression rate: 0.64. In the PLS patient group, the mean scores were the following: mean UMN score: 14; mean ALS∖FRS rate: 33.5; mean bulbar score: 10.5; mean MRC total score: 117.5; mean disease progression rate: 0.26 (Table 1).

### Stroop RTs

**Figure 1:**
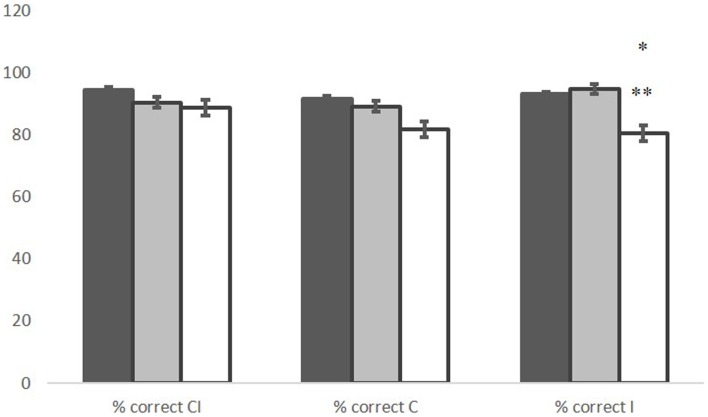
**Percentage of correct responses in the choice and the go/no-go condition, in control (black), PLS (gray), and ALS (white) groups**. *ALS vs. CNT *p* = 0.014; **ALS vs. PLS *p* = 0.05. Line bars over each column indicate Standard Error.

The MANOVA revealed significant group effects in the percentage of correct responses (*F* = 5.057, *p* = 0.003). In the go/no-go I condition, ALS patients committed significantly more errors than controls (*p* = 0.014) and PLS patients (*p* = 0.05). No significant difference was found between PLS patients and controls (Figure 1).

**Figure 2:**
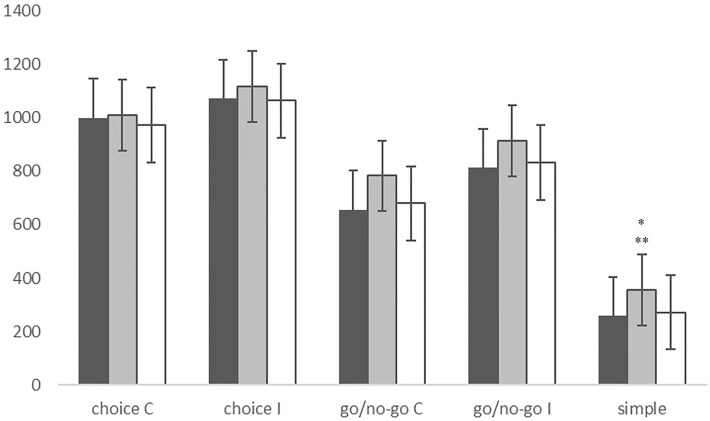
**Reaction times in the choice condition, the go/no-go condition and the simple reaction time condition, in CNT subjects (black), PLS patients (gray), and ALS patients (white)**. *PLS vs. CNT *p* < 0.001; **PLS vs. ALS *p* = 0.002. Line bars over each column indicate Standard Error.

The MANOVA also revealed significant group effects in RTs (*F* = 5.437, *p* < 0.001). In fact, PLS patients appeared significantly slower than controls (*p* < 0.001) and ALS patients (*p* = 0.002) in the SRT condition. ALS patients instead showed no significant differences in RTs compared with controls (Figure 2). RT was negatively correlated with bulbar score in the go/no-go I condition (ρ = −0.455, *p* = 0.008) and disease progression rate in the simple RT condition (ρ = −0.357, *p* = 0.038).

### ERPs latency

**Figure 3:**
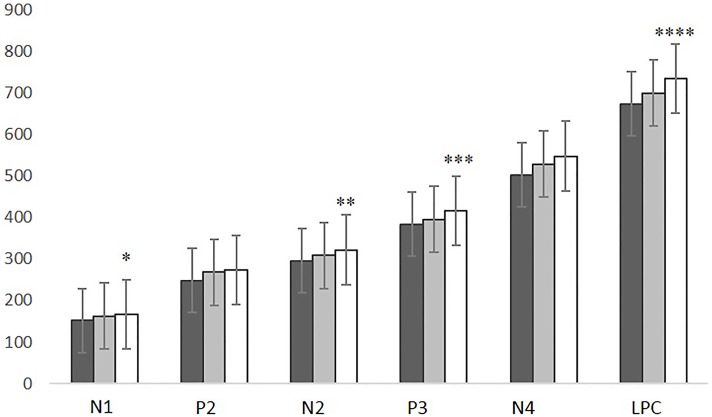
**N1, P2, N2, P3, N4, and LPC latencies in CNT subjects (black), SLP patients (gray), and ALS patients (white)**. ALS vs. CNT: *p = 0.021; **p = 0.043; ***p = 0.036; ****p = 0.004. Line bars over each column indicate Standard Error.

The MANOVA revealed a significant group effect in ERP latencies (*F* = 3.627, *p* = 0.004). N1, N2, P3, and LPC latencies were significantly delayed in ALS patients compared with controls (N1: *p* = 0.021; N2: *p* = 0.043; P3: *p* = 0.036; LPC: *p* = 0.004). No latency differences were found between PLS patients and controls or between ALS and PLS patients (Figure 3). A positive correlation was found between N4 latency and disease duration (*r* = 0.446, *p* = 0.006).

### ERPs amplitude and topography

**Figure 4:**
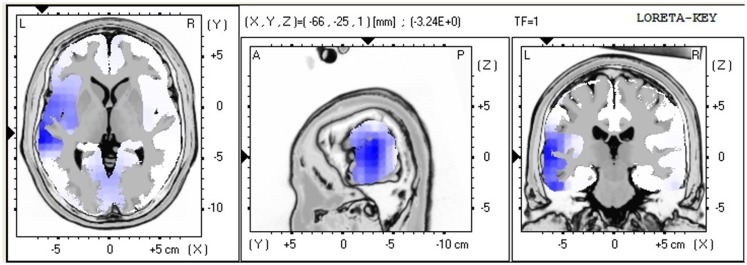
**LORETA non-parametric voxel-wise comparison maps between ALS and control groups in the P2 time window**. Blue: significantly lower activity in ALS.

**Figure 5:**
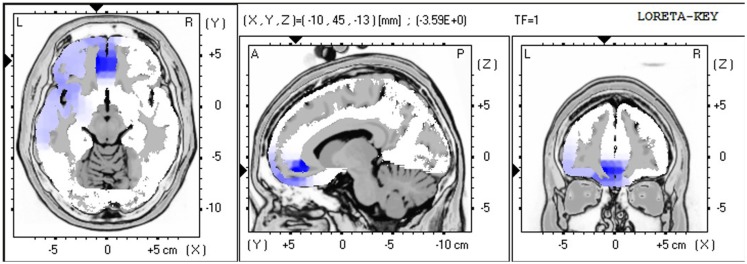
**LORETA non-parametric voxel-wise comparison map between ALS and control groups in the P3 time window**. Blue: significantly lower activity in ALS.

**Figure 6:**
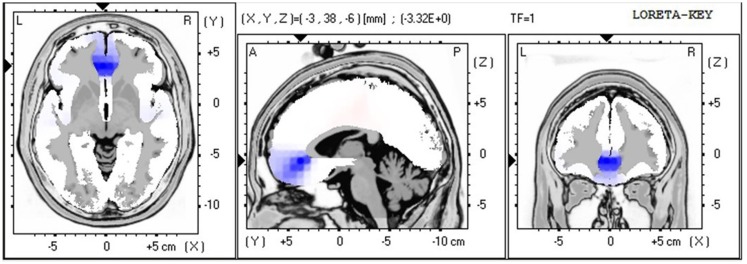
**LORETA non-parametric voxel-wise comparison map between ALS and control groups in the N4 time window**. Blue: significantly lower activity in ALS.

The LORETA non-parametric voxel-wise analysis revealed significant group differences. In the P2 time window (Figure 4), the ALS group exhibited significantly decreased activation of the left superior and middle temporal gyri compared with controls. In the P3 (Figure 5) and N4 (Figure 6) time windows, ALS patients exhibited significantly reduced activation of the ACC and medial frontal gyrus compared with controls. No differences were found between PLS patients and controls.

## Discussion

The aim of the present study was to explore differences in executive function in ALS and PLS patients. We investigated RT performance and bioelectrical activity during a covert Stroop test in non-demented patients with ALS and PLS. ALS patients had significantly lower accuracy in the execution of the task but were not slower, whereas PLS patients did not show reduced accuracy, despite a significant slowing of RTs. Moreover, ALS patients but not PLS patients presented significantly delayed ERP components compared with controls. Reaction time speed but not ERP latency were correlated with clinical scores. Voxel-wise group comparisons of ERPs using LORETA showed decreased frontotemporal activity in the P2, P3, and N4 time windows in ALS patients compared with controls.

### Reaction times

The reduced RT accuracy found in ALS patients, who committed significantly more errors than the other two groups, suggests that conflict-monitoring function, which is necessary for processing competing information and is supposedly mediated by the ACC (Botvinick et al., 1999, 2001), was impaired in ALS patients. Our RT findings are consistent with previous studies that reported executive function deficits in ALS (Abrahams et al., 1997, 2000; Goldstein et al., 2011; Phukan et al., 2012).

The RT results in PLS patients were not consistent with previous neuropsychological studies, which reported impaired performance in PLS patients in a subgroup of tasks used for neuropsychological assessment. Functional frontal lobe impairments appeared to be similar to cognitive dysfunction in ALS (Caselli et al., 1995; Piquard et al., 2006; Grace et al., 2011). In the present study, PLS patients were significantly more accurate than ALS patients, and their RT motor performance was slower, which was expected when considering motor symptoms, but qualitatively similar to healthy controls in terms of accuracy. However, in these previous studies, the subjects had to emit a vocal or motor response, and the results cannot be considered completely independent from movement deficits. Therefore, motor impairments may at least partially account for the inconsistency with our present results.

### ERPs latency

Amyotrophic lateral sclerosis patients presented significant delays in several Stroop ERP components compared with controls, mainly in the N1, N2, P3, and LPC components. N1 is assumed to reflect selective attention to basic stimulus characteristics, initial selection for later pattern recognition, and intentional discrimination processing (Vogel and Luck, 2000). Its source is located in the inferior occipital lobe, occipito-temporal junction (Hopf et al., 2002), and inferior temporal lobe (Bokura et al., 2001). The N2 component in go/no-go-like tasks has been attributed to response inhibition mechanisms (Jodo and Kayama, 1992; Gonzalez-Rosa et al., 2013). However, the N2 component has also been reported to occur in relation to covert responses in the present study and previous studies (Pfefferbaum et al., 1985). This would indicate that it is not completely attributable to the inhibition of responses and that it may at least partially account for conflict monitoring. N2 is especially pronounced over the fronto-central electrodes and has been proposed to reflect ACC sensitivity to conflict (van Veen and Carter, 2002).

The P3 component is elicited in tasks related to stimulus differentiation and appears when a memory representation of the recent stimulus context is updated upon the detection of deviance from it (Sutton et al., 1965). The frontal P300 component in go/no-go-like tasks has been associated with an inhibitory mechanism (Gajewski and Falkenstein, 2013). However, in the present study, the subjects only had to mentally discriminate between congruent and incongruent stimuli; therefore, conflict did not arise at the response level. Thus, the P3 component observed herein most likely reflects the detection of conflict that arose at the level of the semantic encode.

The LPC component is extended positivity that peaks 600–700 ms after stimulus onset and is attributable to the semantic processing of word meaning (Liotti et al., 2000). The ERP delay observed in the ALS group is consistent with previous studies that used oddball paradigms (Hanagasi et al., 2002; Paulus et al., 2002; Ogawa et al., 2009). Altogether, our RT and ERP findings reflect cognitive control impairment in ALS patients.

Primary lateral sclerosis patients showed no abnormalities in ERP latency, despite the important slowing of RTs. This result is only apparently in opposition to the RT results, in which lower accuracy without slowing was observed in ALS patients, and a delay but normal accuracy was observed in PLS patients. In fact, slower RTs in tasks that require a motor response in PLS patients appear to be mainly related to movement impairments, as suggested by their normal frequency of correct responses.

Moreover, the lack of cognitive processing abnormalities in PLS, particularly with respect to ALS, unlikely depends on differences in disease duration because this feature would have favored the ALS group in our sample.

### Correlations

Clinical scores that assess upper and lower motor neuron impairment were correlated with RTs but not with ERP latencies, which were delayed in ALS. This finding allows us to disentangle cognitive and movement impairments that contribute to task performance. Mental discrimination of the Stroop stimuli accounted for cognitive processing, independent of motor performance, suggesting that this version of the task may be useful for cognitively assessing patients in advanced stages of the disease when profound motor disability interferes with communication or leads to a virtual *locked-in* syndrome. Furthermore, this approach may be useful for evaluating eligibility for brain-computer interface. Finally, computerized RTs, which provide useful measures in the assessment of executive function, may reflect the severity of motor impairment as indicated by their correlation with clinical assessment.

### ERPs amplitude and topography

In ALS patients compared with controls, the voxel-wise group comparison of ERPs using LORETA showed a significant decrease in the activation of the left superior and middle temporal gyri in the P2 time window, suggesting impaired lexical processing, and a significant decrease in activity in the ACC and medial frontal gyrus in the P3 and N4 time windows, suggesting impaired conflict monitoring.

The findings of decreased activity in our sample of ALS patients are consistent with previous ERP studies that reported hypoactivity in frontotemporal regions in ALS patients (Vieregge et al., 1999; Hanagasi et al., 2002; Hammer et al., 2011), resulting in impaired performance in tasks that rely on executive function.

Our results are also consistent with previous studies (Abrahams et al., 2000, 2004) that reported reduced verbal fluency that relies on executive dysfunction in ALS patients. The authors found that language dysfunction also plays a role in word retrieval deficits in ALS patients, reflected by hypoactivity in the middle and superior temporal gyri, which are involved in lexical and semantic processing (Abrahams et al., 2004). Our findings also suggested impaired lexical processing in ALS patients, reflected by decreased activation of the left superior and middle temporal gyri in the P2 time window, which appeared to be independent of executive dysfunction, reflected by decreased activity in the ACC and medial frontal gyrus in the P3 and N4 time windows.

Our findings appear to be partially inconsistent with Goldstein et al. (2011), who reported increased activity in these areas during the performance of the classic Stroop task but a decrease during a modified version of the same task. However, these two studies are only partially comparable. In fact, in the study by Goldstein et al. the subjects had to name the color of the ink in which the stimuli were presented. To provide the correct response, the subjects had to inhibit the automatic reading of the incongruent color word. Thus, the results reported account for altered inhibitory processing in ALS.

In the present study, mental discrimination was used to avoid movement or speech impairment contamination of the results. The subjects simply had to detect stimulus conflict that arose only at the level of semantic encoding, which contributes to overall interference in the classic Stroop task, together with the response level conflict (Milham et al., 2001; van Veen and Carter, 2005). The subjects also had to discriminate between congruent and incongruent stimuli without generating any response. Thus, our results more likely suggest impaired conflict-monitoring function in ALS patients.

In PLS patients compared with controls, the voxel-wise group comparison of ERPs using LORETA did not indicate differences in activity, in contrast to the findings in ALS patients. This result is consistent with the ERP and RT results.

Further activation studies that directly compare ALS and PLS patients are needed to better clarify differences in cognitive function between these two MNDs.

Previous studies compared cerebral involvement in ALS and PLS patients (Ciccarelli et al., 2009) and found a reduction of FA in ALS patients in white matter adjacent to the superior frontal gyrus. In PLS patients, they found lower FA in the body of the corpus callosum and white matter adjacent to the right primary motor cortex. These results, although not directly comparable with our results, suggest the possibility that ALS and PLS present a different pattern of cerebral involvement, which is consistent with our present findings.

## Conclusion

Our aim was to identify psychophysical and neurophysiological features able to characterize ALS and PLS with respect to frontal involvement. Although beyond our scope, the present study lacks a formal and comprehensive neuropsychological testing for correlation and comparison with our data. Further studies are needed specifically addressing the comparative value of neuropsychological and psychophysiological approaches in the assessment of people with MND.

In conclusion, the present results suggest a different extent of cognitive involvement in ALS compared with PLS. ALS is increasingly recognized as a multisystem disorder that presents a spectrum of executive and behavioral impairments that reflect frontal dysfunction. In contrast, PLS appears to spare cognitive function and manifests predominantly with motor symptoms. Our results suggest the possibility that the covert version of the Stroop task used in the present study, which involves the mental discrimination of stimuli and not vocal or motor responses, may be useful for assessing cognitive function in ALS patients in advanced stages of the disease when other cognitive tasks are not applicable. It may also help in evaluating eligibility for brain-computer interface.

## Conflict of Interest Statement

The authors declare that the research was conducted in the absence of any commercial or financial relationships that could be construed as a potential conflict of interest.
